# Longer-term outcomes of gastroesophageal reflux disease treated with magnetic sphincter augmentation

**DOI:** 10.1093/dote/doad014

**Published:** 2023-03-20

**Authors:** Aiysha Puri, Sue Steven, Sheraz R Markar, Nicholas Boyle

**Affiliations:** Department of Surgery and Cancer, Imperial College London, London, UK; Department of Surgery, Reflux UK, London, UK; Department of Surgery and Cancer, Imperial College London, London, UK; Department of Molecular Medicine and Surgery, Karolinska Institutet, Stockholm, Sweden; Nuffield Department of Surgery, University of Oxford, Oxford, UK; Department of Surgery, Reflux UK, London, UK

**Keywords:** anti-reflux surgery, diseases of the esophagus, gastroesophageal reflux (GERD), health-related quality of life

## Abstract

Surgical intervention for gastroesophageal reflux disease (GERD) has historically been limited to fundoplication. Magnetic sphincter augmentation (MSA) is a less invasive alternative that was introduced 15 years ago, and it may have a superior side-effect profile. To date, however, there has been just a single published study reporting outcomes in a UK population. This study reports quality-of-life (QOL) outcomes and antacid use in patients undergoing MSA, with a particular focus on postoperative symptoms and those with severe reflux. A single-center cohort study was carried out to assess the QOL outcomes and report long-term safety outcomes in patients undergoing MSA. GERD-health-related quality of life (GERD-HRQL) and Reflux Symptom Index (RSI) scores were collected preoperatively, and immediately postoperatively, at 1-, 2-, 3-, and 5-year follow-up time points. All patients underwent preoperative esophagogastroduodenoscopy, impedance, and manometry. Two hundred and two patients underwent laparoscopic MSA over 9 years. The median preoperative GERD-HRQL score was 31, and the median RSI score was 17. There was a reduction in all scores from preoperative values to each time point, which was sustained at 5-year follow-up; 13% of patients had a preoperative DeMeester score of >50, and their median preoperative GERD-HRQL and RSI scores were 32 and 15.5, respectively. These were reduced to 0 at the most recent follow-up. There was a significant reduction in antacid use at all postoperative time points. Postoperative dilatation was necessary in 7.4% of patients, and the device was removed in 1.4%. Erosion occurred in no patients. MSA is safe and effective at reducing symptom burden and improving QOL scores in patients with both esophageal and laryngopharyngeal symptoms, including those with severe reflux.

## INTRODUCTION

Gastroesophageal reflux disease (GERD) is the most common disease of the esophagus and affects about 10% of the western population.^1^^,^^2^ Treatment options for most include lifestyle changes and medical acid suppression therapy. However, despite maximal medical therapy, nearly 40% of patients can experience persistent symptoms,^3^^,^^4^ and up to 90% of patients fail medical management.^5^ In these patients, laparoscopic fundoplication is considered as the ‘gold standard’ surgical option. However, only 1% of patients who could potentially benefit from fundoplication undergo surgery, despite surgical enthusiasm and an evidence base dating back decades. Contributory factors include fear of the side effects following surgery, such as gas bloat^6^ and an inability to belch or vomit,^7^ as well as anatomic failure of the repair. Markar et al.^8^ reported that, on the basis of community data in the United Kingdom, PPI usage had resumed in 59.4% of post-fundoplication patients within 10 years and that, in 9.4% of post-fundoplication patients, surgical reintervention was necessary.

When magnetic sphincter augmentation (MSA) was successfully introduced in 2007, the original concept was that it would fit into the treatment options for GERD as a less invasive alternative option than laparoscopic fundoplication.^9^ It entails laparoscopic placement of titanium beads with a magnetic core around the lower esophageal sphincter (LES); it augments the physiological barrier and preserves gastric anatomy using a standardized and easily reproducible technique; and it is more easily reversed than fundoplication. Short-term outcomes initially demonstrated no intraoperative complications,^9^^,^^10^ in addition to an improvement in patient-reported health-related quality of life (HRQL) outcomes, which have been sustained at long-term follow-up.^11^ A recent meta-analysis that compared MSA to fundoplication in 1099 patients again demonstrated the safety of the device and, importantly, demonstrated that it is as effective as fundoplication in controlling symptoms of GERD^12^ and that MSA may be associated with a reduced risk of gas bloating compared to fundoplication. Patients undergoing anti-reflux surgery primarily are choosing to have this procedure to improve their symptoms, and therefore, an improvement in symptoms and a reduction in HRQL scores should be the primary aim.

The manufacturers of the LINX device used for MSA report that nearly 40,000 patients have now undergone sphincter augmentation worldwide, and yet adoption has varied geographically. Regulatory approval was granted in the United Kingdom in 2012 (NICE IPG431), and yet there has been only a single series reporting outcomes in just 48 patients over 3 years.^13^

The aim of this study was to report the largest series of patients undergoing MSA in the United Kingdom, with a particular focus on HRQL outcomes, antacid dependency, operative outcome measures, and patients with severe reflux.

## METHODS

This was a single-center cohort study, from a prospectively maintained database, to assess the effectiveness of the MSA in the management of patients with GERD. All patients undergoing MSA implantation between 2012 and August 2020 were included. Patients were referred having failed medical management. Patients eligible for either a Nissen’s fundoplication or MSA were comprehensively consented; however, the decision regarding surgical approach was determined by patient choice where both procedures were deemed surgically appropriate. Patients were only excluded if they had previously undergone anti-reflux surgery in the past. MSA was introduced within the standard clinical governance framework at this center.

When initially introduced, the manufacturers and the FDA suggested several precautions for use, including the presence of hiatus hernias larger than 3 cm,^14^^,^^15^ Barrett’s esophagus, severe GERD as reflected in grades C/D esophagitis, and poor esophageal motility, defined as a mean distal amplitude of <32 mmHg. These precautions were purely advisory and have been subsequently withdrawn.^16^^,^^17^ With experience, the authors have offered MSA to all patients as an alternative to fundoplication except for those with type III hiatus hernias and ineffective motility as defined above.

As part of the preoperative pathway, patients routinely received esophagogastroduodenoscopy (EGD), pH, and manometry testing and were administered GERD-HRQL and Reflux Symptom Index (RSI) questionnaires and were questioned regarding their use of antacids preoperatively and at 6 months, 1 year, 2 years, 3 years, and 5 years postoperatively where applicable. A total quality-of-life (QOL) score was created by adding the total scores of the GERD-HRQL and the RSI questionnaire.

At endoscopy, the size of the hiatus hernia, the presence of esophagitis, and the presence of Barrett’s esophagus were determined. Patients either underwent pH testing via trans-nasal pH testing or via the Bravo wireless system. At manometry testing, the lower-esophageal sphincter pressures, distal contractile integral were collated in addition to a general comment about esophageal motility.

The procedure was carried out as a laparoscopic procedure as previously described,^10^ all patients underwent a formal hiatal dissection, and crural repair. The implant was sized using the bespoke sizing device and clinical assessment with a minimum of two clicks above the ‘popping’ of the device.

Continuous data are reported as the median and interquartile range (IQR), or the mean and standard deviation (SD). Patients were their own control and were compared to preoperative results. Descriptive analysis, followed by paired *t*-tests, was performed using GraphPad Prism version 8.0.0 for Mac, GraphPad Software, San Diego, California, USA. A *P-*value of <0.05 was considered to be statistically significant.

### Clinical outcomes

The primary outcome was the assessment of QOL in patients receiving MSA, with particular focus on regurgitation, dysphagia, and gas bloating symptoms, in addition to assessing use of antacids. Furthermore, QOL outcomes were interrogated in patients with severe reflux. The secondary outcomes were assessment of short- and long-term outcomes associated with MSA.

### QOL assessment tools

The GERD-HRQL is a tool employed to assess reflux symptoms and has been validated across many countries and in many languages.^18^ It comprises a 11-question self-administered questionnaire where the score ranges from 0 to 5, and a total score of 50, and a total score of >15 is considered to be abnormal.^19^ The RSI comprises seven questions, where the score ranges from 0 to 5; it has been demonstrated to be effective when considering patients with laryngopharyngeal reflux (LPR) disease both preoperatively and postoperatively, and a score of ≥13 is considered to be indicative of LPR.^20^

### Severe reflux

Severe reflux was defined as a DeMeester score of >50, a cut-off used by other such papers.^16^

## RESULTS

Over a 9-year study period, 202 patients underwent placement of the LINX device. 61% (*n* = 124) were male, and the mean age at procedure was 48 years (range: 18–80 years) (Table 1). Most patients had no significant comorbidities (*n* = 148), and the most common comorbidity was hypertension in 6% (*n* = 13), which was followed by respiratory conditions in 5% (*n* = 11); 47 (23%) patients had undergone previous abdominal surgery. Over the same time period, 73 patients under went laparoscopic fundoplication, 54% (*n* = 39) of which were carried out in the first 3 years of the study period.

**Table 1:** Preoperative demographics

	*n =* 202
Gender M F	124 78
Mean age at procedure (SD)	48 (18.4)
Mean years of GERD symptoms (SD)	18.4 (113.9) (*n* = 174)
Mean years of antacid use (SD)	9.8 (52.6) (*n* = 140)
Dysphagia present (%)	87 (43) (*n* = 198)
Reflux Symptom Index $\ge$13 (%)	125 (62)
Median hiatal hernia size (cm)	2 (0–8)
Median DeMeester score (IQR)	23.2 (23.9–38.2)
Mean LES pressure (SD) (mmHg)	18.07 (14.07)
Mean distal esophageal amplitude (SD) (mmHg) (range)	54.41 (26.21) (13.8–182.00)
Mean % total time pH < 4	6.6 (8.5)
Comorbidities 0 1 2 3	147 38 14 2
Previous abdominal surgery	47 (*n* = 175)

GERD, gastroesophageal reflux disease; IQR, interquartile range; LES, lower esophageal sphincter; SD, standard deviation.

### Preoperative

The mean number of years of GERD symptoms was 18.4, and the mean number of years of antacid use was 9.8. Dysphagia was present in 43% of patients (*n* = 87). The median hiatal hernia size was 2 cm at EGD (range: 0–8 cm). Patients reported a variety of symptoms, and the most commonly reported symptom was ‘retrosternal burning’ in 60 patients (29.7%), which was followed by dyspepsia in 36 (17.8%) and by cough and retrosternal discomfort in 18 each, respectively (8.9%).

### Physiology testing

On preoperative impedance testing, the median DeMeester Score was 23.2 (IQR: 23.9–38.2), and in 13.4% (*n* = 27), the DeMeester score was ≥50. BRAVO capsules were utilized in 11 patients in lieu of trans-nasal pH impedance testing. The mean acid exposure time was 6.6% (SD: 8.5%) (normal as per the Lyon consensus <4%^21^).

The mean LES pressure was 18.07 mmHg (SD: 14.07 mmHg) (normal range: 10–45 mmHg), and the mean distal esophageal amplitude was 54.41 mmHg (SD: 26.21 mmHg).

### Follow-up

Patients were routinely discharged from clinical care at 6 months following the operation if they were symptom-free, and they were contacted at 1 year, 2 years, 3 years, and 5 years postoperatively to obtain symptom scores. Median follow-up was 2 years (IQR: 1–3). All patients were eligible for follow-up at 1 year following the operation, and HRQL scores were obtained from 80% of patients (Table 2); 184 patients were eligible for follow-up at 2 years and 3 years following the operation, and data were available in 68% and 41% of patients, respectively; 88 patients were operated on ≥5 years ago, and in this population, 38 provided HRQL scores (43%).

**Table 2:** Follow-up data availability

	Number of patients eligible	Number of patients completed QOL scores (%)
Pre-op	202	199 (99)
Post-op 6 months	202	181 (90)
Post-op 1 year	202	162 (80)
Post-op 2 years	184	126 (68)
Post-op 3 years	184	75 (41)
Post-op 5 years	88	38 (43)

QOL, quality of life.

### Primary outcomes

#### QOL scores

The GERD-HRQL and RSI scores were combined to give a total score; preoperatively, the median total QOL score was 44.5 (IQR 32-63), the median GERD-HRQL score was 31 (IQR 16-39), and the median RSI Score was 17 (IQR 9-25). The RSI was >13 in 62% of patients preoperatively (*n* = 125).

**Fig. 1:**
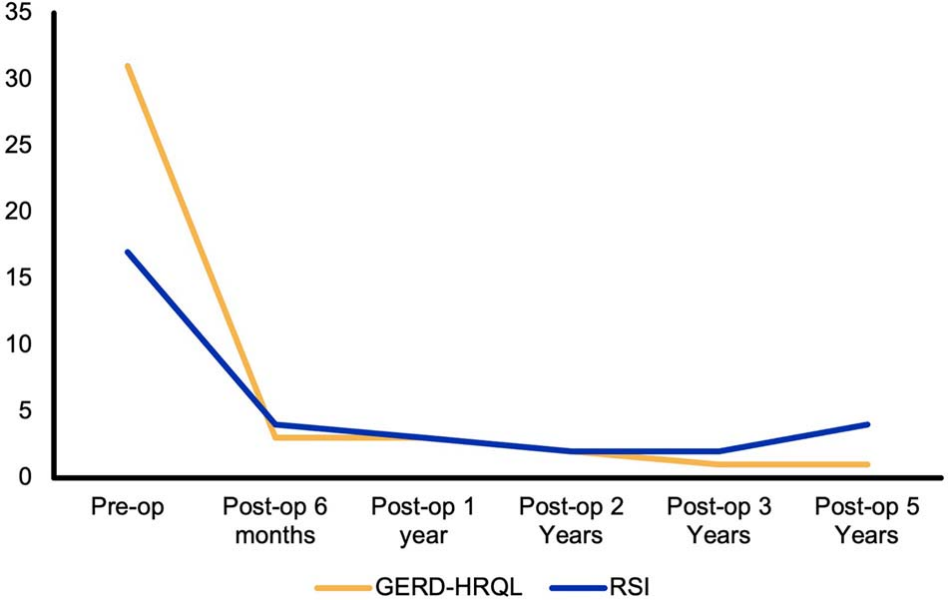
Median GERD-HRQL and RSI scores over 5-year follow-up. GERD, gastroesophageal reflux disease; HRQL, health-related quality of life; RSI, Reflux Symptom Index.

**Fig. 2:**
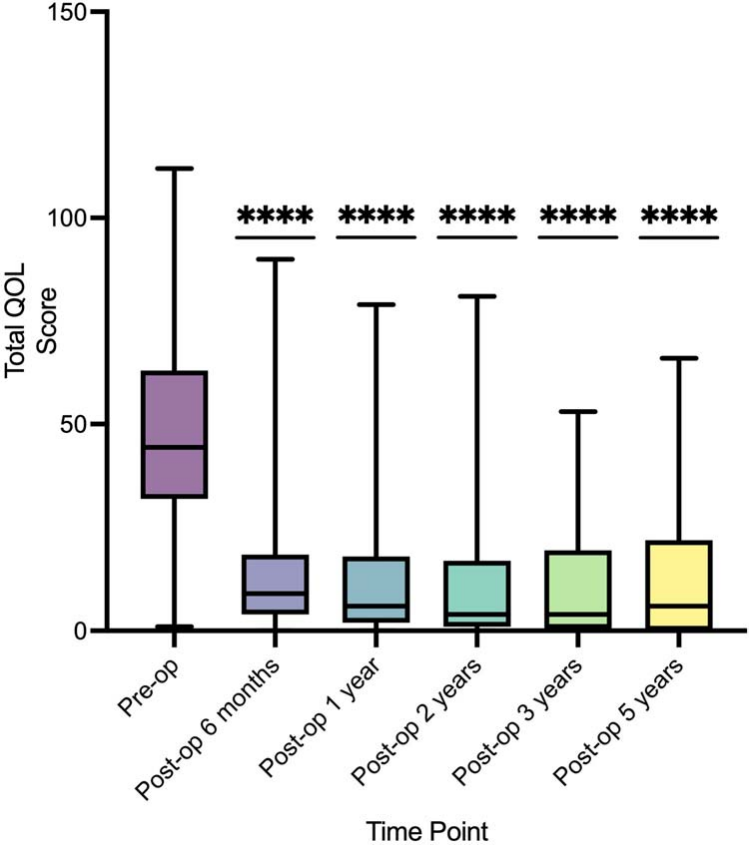
Total QOL scores over 5-year follow-up. (Reduction demonstrated compared to preoperative score, ^*^^*^^*^^*^  *P* ≤ 0.0001.). QOL, quality of life.

There was a reduction in all scores from preoperative values to each time point that was sustained at 5-year follow-up (Fig. 1). The median GERD-HRQL score at the latest follow-up was 2 (IQR: 0–9), and this was a significant reduction compared to the preoperative value of 31, with a *P-*value of <0.0001. The total QOL score was significantly lower in patients all time points compared to preoperatively; at 6-month follow-up, this was significant to *P* < 0.0001 using a paired *t*-test (95% CI: −35.96 to −29.34) (Fig. 2). Overall, there was greater than 50% reduction in total scores compared to preoperatively at each time point (Supplementary Fig. 1).

##### Antacid use

**Fig. 3:**
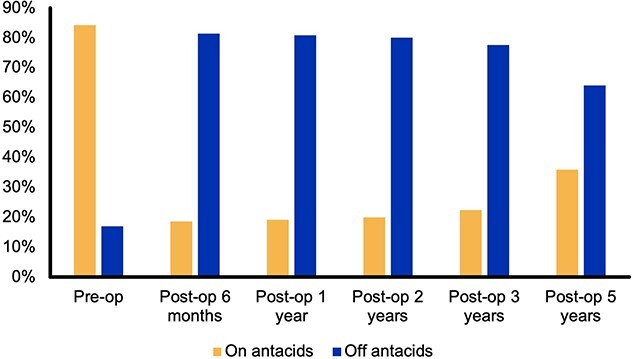
Antacid use over 5-year follow-up.

Preoperatively, 84% (*n* = 149) required antacids to control symptoms; at immediate postoperative follow-up, this was reduced to 19% (*n* = 30), and this was a significant reduction at all follow-up intervals, *P* < 0.0001 (Fig. 3).

#### Symptom breakdown

The quality-of-life questions relating to regurgitation, upper abdominal bloating, flatulence, and dysphagia (sum of the scores of the questions ‘Do you have difficulty swallowing?’ and ‘Do you have pain on swallowing’) were individually analyzed.

#### Abdominal bloating

**Fig. 4:**
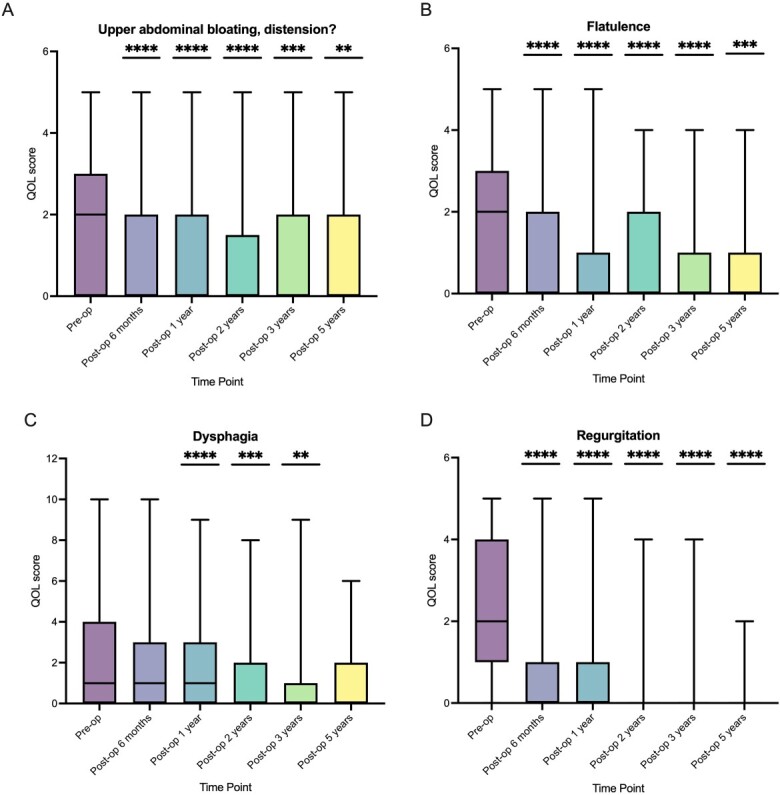
Breakdown of symptom scores over 5-year follow-up. A - upper abdominal bloating, distension, B - flatulence, C - dysphagia, D - regurgitation (Compared to preoperative score, ^*^^*^^*^^*^  *P* ≤ 0.0001, ^*^^*^^*^  *P* ≤ 0.001, ^*^^*^  *P* ≤ 0.01.)

The median preoperative score was 2 (IQR: 0–3), this reduced to 0 at all postoperative time points (Fig. 4A). There was a statistically significant reduction in the scores at all time points compared to preoperatively.

#### Flatulence

The median preoperative score was 2 (IQR: 0–3), and this reduced to 0 at all postoperative time points (Fig. 4B). There was a statistically significant reduction in the scores at all time points compared to preoperatively.

#### Dysphagia

This was assessed using the two following questions: ‘Do you have difficulty swallowing?’ and ‘Do you have painful swallowing?’ which were assessed using a Likert score of 0 (none) to 5 (severe). The median preoperative sum of the scores for questions relating to dysphagia was 1 (IQR: 0–4), and this reduced to 0 after 2 years of follow-up (Fig. 4C). There was a statistically significant reduction in the score compared to pre-op for post-op year 1, post-op year 2, and post-op year 3.

#### Regurgitation

The median preoperative score was 2 (IQR: 1–4), and this reduced to 0 at all postoperative time points (Fig. 4D). There was a statistically significant reduction in the score at all time points compared to preoperatively.

### Secondary outcomes

#### Short term: procedure and recovery

All cases were carried out laparoscopically. In all cases, a circumferential distal esophageal dissection and crural repair were performed. The median device size was 14 beads (Table 3). There were no intraoperative complications. The median length of stay was 0.6 days; where patients stayed overnight, this was due to patients’ preference as patients traveled significant distances to have the procedure carried out. There was one postoperative death in a patient with undiagnosed cardiac disease who suffered from a myocardial infarction. One patient was diagnosed with a deep vein thrombosis and subsequent small volume pulmonary emboli. Four patients required re-admission to hospital postoperatively with fever, chest pain (two), and nausea secondary to transient gastroparesis, but none required operative intervention.

**Table 3:** Operative characteristics

	*n = 202*
Laparoscopic procedure	202
Device size 12 13 14 15 16 17 18	3 72 61 43 16 0 1

#### Long term

In the long term, 15 patients required dilatation of the gastroesophageal junction following insertion of the implant (7.43%), of whom 2 required a second dilatation. Four patients (1.98%) underwent device ex-plantation. Two were removed because of persistent dysphagia which did not improve with dilatations; one was removed due to unexplained back and abdominal pain without dysphagia; and one was removed due to persistent reflux symptoms, and this patient went onto have a Toupet fundoplication which did not improve symptoms. One patient suffered disruption of the device due to manufacturing failure in an early device, and his symptoms were of recurrent GERD following implantation of the device. Two patients had symptomatic recurrence of their hiatal hernia at 1-year follow-up (0.99%). Erosion of the device occurred in no patients.

#### Severe reflux: outcomes in patients with DeMeester score $\mathbf{\ge}$50

In the 27 patients with a DeMeester Score >50, the mean DeMeester score was 90.02 (SD: 58.71).

**Fig. 5:**
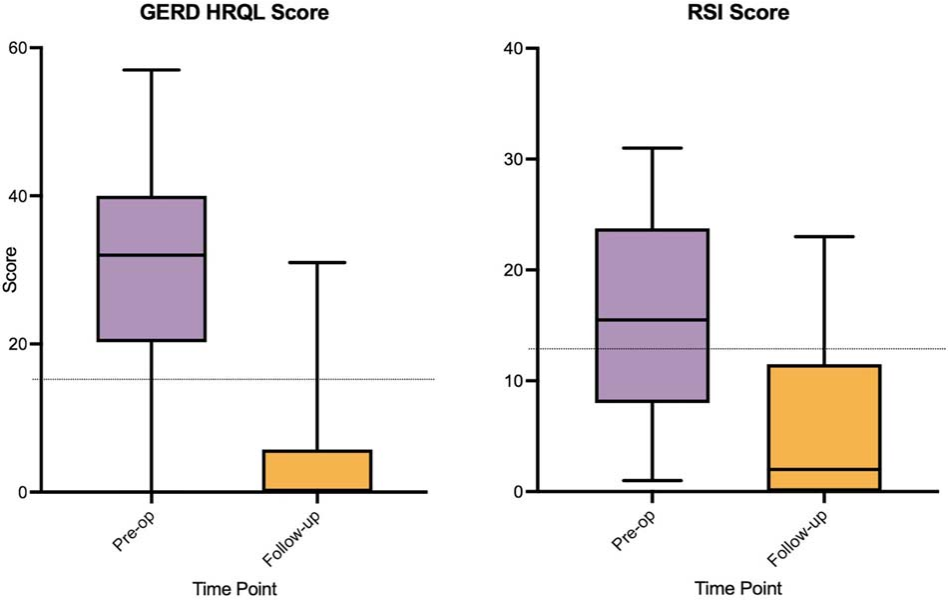
GERD-HRQL and RSI scores in patients with preoperative DeMeester ≥50. GERD, gastroesophageal reflux disease; HRQL, health-related quality of life; RSI, Reflux Symptom Index.

Twenty-four patients with a DeMeester score >50 at preoperative impedance testing had follow-up QOL data available (Fig. 5). The median preoperative GERD-HRQL score was 32 (IQR: 20–40), and this reduced to 0 at the most recent follow-up available (IQR: 0–5.75). The median preoperative RSI score was 15.5 (IQR: 8–23.75), and this reduced to 0 at the most recent follow-up (IQR: 0–11.5).

## DISCUSSION

Insertion of the LINX device to surgically manage reflux is safe and improves patient-reported QOL outcomes. Patient-reported measures of regurgitation and gas bloat are reduced in the postoperative period, and this is sustained where 5-year follow-up is available. GERD-HRQL and RSI scores are reduced postoperatively even in patients with severe reflux. Patients were able to stop or reduce their use of antacids following MSA. Over a period of 5 years, good reflux control was maintained, establishing the durability of this technique, with a low long-term complication rate. In the latter 6 years of the study period in this center, only 35 patients opted to have a laparoscopic fundoplication when compared to 172 MSA procedures carried out.

### Symptom control

Previously published data have demonstrated that sphincter augmentation device has an improved postoperative symptom profile compared to a traditional fundoplication; with MSA, there is reduced bloating, and patients retain the ability to belch.^12^^,^^22^^,^^23^ Our results strongly support that there is a reduction in bloating and abdominal distension in addition to flatulence compared with preoperatively in patients undergoing the MSA, which can affect between up to 40% and 57% of patients undergoing fundoplication as per the LOTUS trial.^6^ The proposed mechanisms to explain the lower rate of surgical re-intervention in MSA are first the preservation of the ability to belch following MSA enabling gastric decompression,^17^ which is in contrast with patients undergoing fundoplication who cannot vent the stomach, which may impact the crural repair in the early stages of recovery.

The study by Warren et al. suggested that there was a higher rate of dysphagia in patients following implantation compared with fundoplication (44% vs. 32%).^24^ The rate of dilatation within the cohort is low, and in keeping what has been reported in previous series.^25^^,^^26^ In their retrospective case series of 380 patients, Ayazi et al. reported that 31% of patients had required at least one dilatation; however, over time, this fell to 18%.^25^ This reduction was attributed to key alterations in clinical practice, namely the re-introduction of a normal diet in the immediate postoperative period and change in the sizing protocol for the device.^11^ It has been our practice to emphasize the importance of swallowing normally; we explain to all our patients that the rationale of resumption of a normal diet is to prevent the formation of a restrictive fibrous capsule around the implant^27^ in the first weeks following surgery, which is in contrast with the instructions given to patients following fundoplication. To emphasize that there should be no mechanical cause for them to be unable to swallow, all patients are asked to eat a sandwich before they leave hospital, as the majority are performed as day case procedures.

Commonly, patients encounter increasing dysphagia at 10–14 days following the operation often require the support of a specialist nurse, who will encourage them to persist with their diet. As emphasized in Ayazi’s study, in most patients, dysphagia will resolve within 3 months.^25^ Our practice has always been to combine a visual albeit subjective assessment during the sizing process in addition to using the measuring device and to oversize rather than undersize. Only three patients underwent implantation of a size 12 device, which has been demonstrated in other series to be associated with an increased risk of dysphagia.^11^ These practices may be contributory factors to minimizing significant dysphagia in this series.

### QOL improvements

Within our patient cohort, there was a significant reduction in GERD-HRQL scores and RSI scores at 5-year follow-up. This is in keeping with previous data, which has suggested that MSA is effective in reducing patient-reported QOL scores.^10^^,^^11^^,^^24^ This despite a patient population with more symptomatic reflux; in our cohort, the mean GERD-HRQL preoperative score was 29 when compared with a mean preoperative score of 19, as reported by Ferrari et al.,^11^ and the symptom profile is further supported by the lengthy duration of which antacids had been taken for preoperatively.

### Severe reflux

In the 27 patients in whom the preoperative DeMeester scores were >50, there has been an objective and sizable reduction in both QOL indices. This further supports the evidence that MSA is not only safe in patients with severe reflux but is also effective at controlling symptoms.^16^

### Antacid use

Antacid use was reduced in patients undergoing MSA at all follow-up time points. This is as in other series, and this fits with the evidence to suggest that MSA can improve the DeMeester scores and normalize pH testing.^28^^,^^29^ At 5-year follow-up 64% of our patients were not taking antacids, which is supported by other studies,^30^ and this is lower than in patients in the United Kingdom undergoing fundoplication.^8^

### Patient safety

Previous papers have cited that the MSA is associated with an erosion risk of between 0.1% and 0.3%,^26^^,^^27^^,^^31^ and all of these papers suggest that there is a link between device size and the rate of erosion and development of the laparoscopic sizing tool in 2013 has changed the way in which the device has been fitted. Within our series, there have been no patients that have experienced erosion following implantation. No patients had to undergo a re-do procedure.

The explanation of 1.4% in this series is lower than previously reported series^11^^,^^32^ and is lower than reported rates of revision in laparoscopic fundoplication of approximately 9%.^7^^,^^8^

### Lessons learnt

Since its introduction, the surgical technique for anti-reflux surgery has evolved. The original description of the technique did not include formal circumferential distal esophageal mobilization or crural repair. However, there is a long history of surgical learning going back to Allison,^33^ which has reflected the importance of these maneuvers in preventing the recurrence of hiatal hernias and symptomatic reflux. Furthermore, when measured manometrically during surgery, crural repair and fundoplication appear to contribute equally to the pressure and length of the reconstructed LES mechanism.^34^ Two studies have also reported better symptomatic outcomes in patients undergoing formal esophageal dissection and crural repair when carried out with MSA compared with MSA alone.^35^^,^^36^ The author’s approach has been always to include both esophageal dissection and mobilization to ensure good infra-diaphragmatic esophageal length and subsequent cruroplasty. It maybe that this approach contributed to the low incidence of recurrent reflux symptoms and re-intervention rate of <2% reported in this series.

Finally, it has always been the author’s protocol to place the implant just above the gastroesophageal junction. Although to our knowledge, there is no published evidence that, following surgical mobilization, the esophagus will naturally shorten it seems sensible to assume that this may well be the case. Consequently, more cephalic implantation may increase the likelihood of proximal migration and consequently increase the risk of recurrent reflux symptoms or dysphagia.

### Limitations

A limitation is the lack of comparison with other surgical interventions such as laparoscopic fundoplication. While weight can be a common confounding variable with reflux, we did not monitor the patient’s weight before or after MSA. Data regarding the indication for postoperative use of antacids were unavailable, which is a common limitation of anti-reflux surgery studies.

The main limitation of our study is primarily the retrospective reviewing of patient case notes and the difficulty in following up patients to obtain QOL scores after discharge from routine clinical care (Table 2); this is in spite of making efforts to contact all patients who had incomplete QOL scores. Despite this, at a 2-year follow-up, these data suggest that MSA provides equivalent symptom control as laparoscopic fundoplication^37^ with a better postoperative symptom profile.^6^^,^^7^

### Conclusions

Currently there are no randomized control trial comparing MSA with fundoplication, there have been matched retrospective analyses that have been carried out that demonstrated equipoise in the GERD-HRQL scores and better postoperative results with regard to ability to belch and bloating with MSA.^37^ The data in our series support this.

Further work must entail a randomized control trial to compare the use of MSA with laparoscopic fundoplication in order to demonstrate efficacy, safety, and improvement in patient-reported QOL outcomes.

## DATA AVAILABILITY STATEMENT

The data that support the findings of this study are not openly available due to patient confidentiality and are available from the corresponding author upon reasonable request.

## Supplementary Material

Supplementary_Figure_1_doad014

## Notes

This paper is not based on a previous communication to a society or meeting.

## Notes

Specific author contributions: All authors were involved in conception and design of the study; in addition, all authors were involved in initially drafting the article, and all authors were equally involved in revising it critically for important intellectual content. All authors gave final approval of the version to be published.

## Notes

Financial support: None.
