# Supplementary figures and images for: Longer-term outcomes of gastroesophageal reflux disease treated with magnetic sphincter augmentation
Source: Dis Esophagus. 2023 Mar 20;36(10):doad014. doi: 10.1093/dote/doad014 (PMC10789235; doi:10.1093/dote/doad014)

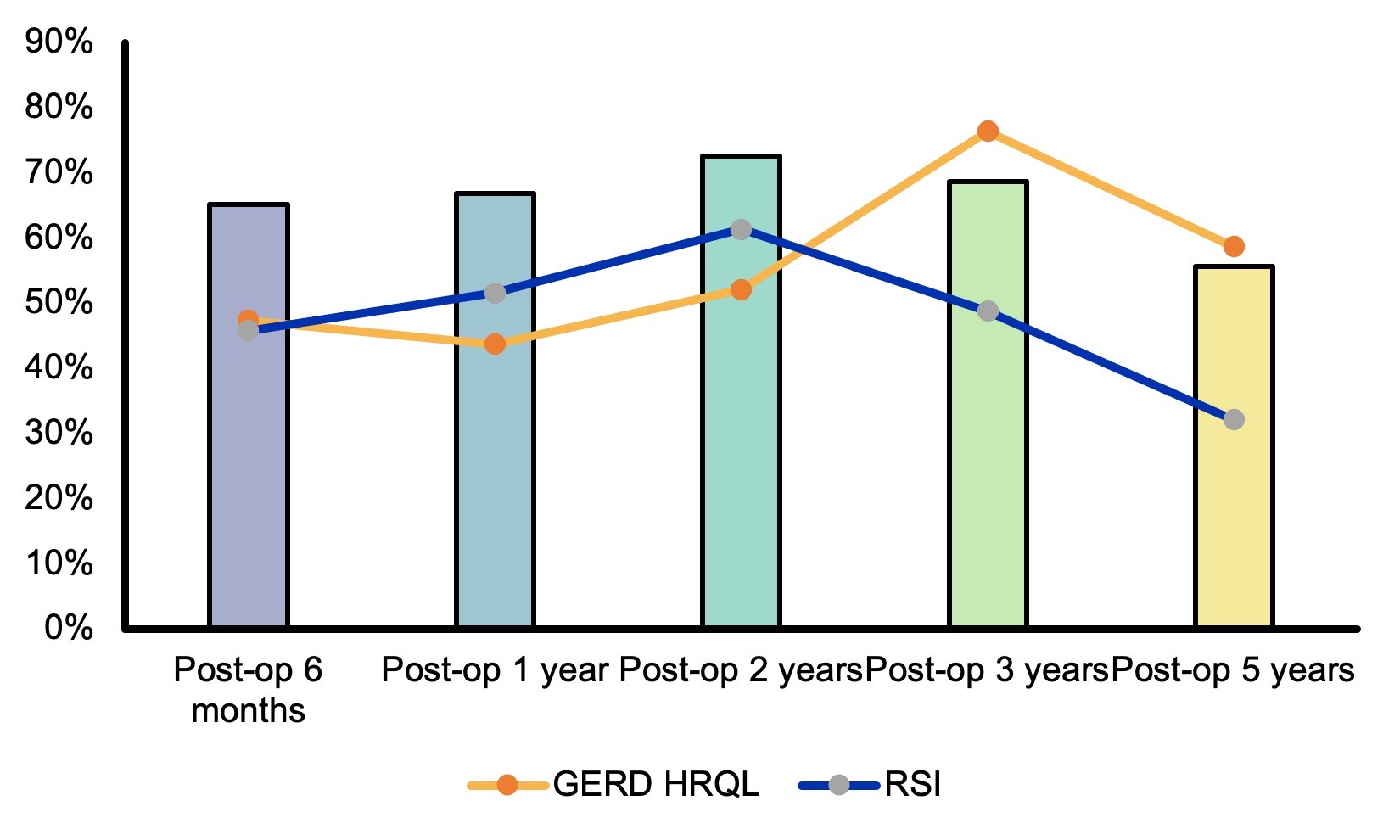

Supplement: Supplementary_Figure_1_doad014 [file supplementary_figure_1_doad014.jpeg]
